# Modified cell cycle status in a mouse model of altered neuronal vulnerability (slow Wallerian degeneration; *Wld*^*s*^)

**DOI:** 10.1186/gb-2008-9-6-r101

**Published:** 2008-06-20

**Authors:** Thomas M Wishart, Helen N Pemberton, Sally R James, Chris J McCabe, Thomas H Gillingwater

**Affiliations:** 1 Centre for Integrative Physiology, University of Edinburgh Medical School, Edinburgh, EH8 9XD, UK; 2 Centre for Neuroscience Research, University of Edinburgh Medical School, Edinburgh, EH8 9XD, UK; 3 Division of Medical Sciences, Institute of Biomedical Research, University of Birmingham, Birmingham, B15 2TH, UK

## Abstract

**Background:**

Altered neuronal vulnerability underlies many diseases of the human nervous system, resulting in degeneration and loss of neurons. The neuroprotective slow Wallerian degeneration (*Wld*^*s*^) mutation delays degeneration in axonal and synaptic compartments of neurons following a wide range of traumatic and disease-inducing stimuli, providing a powerful experimental tool with which to investigate modulation of neuronal vulnerability. Although the mechanisms through which *Wld*^*s *^confers neuroprotection remain unclear, a diverse range of downstream modifications, incorporating several genes/pathways, have been implicated. These include the following: elevated nicotinamide adenine dinucleotide (NAD) levels associated with nicotinamide mononucleotide adenylyltransferase 1 (*Nmnat1*; a part of the chimeric *Wld*^*s *^gene); altered mRNA expression levels of genes such as pituitary tumor transforming gene 1 (*Pttg1*); changes in the location/activity of the ubiquitin-proteasome machinery via binding to valosin-containing protein (VCP/p97); and modified synaptic expression of proteins such as ubiquitin-activating enzyme E1 (Ube1).

**Results:**

*Wld*^*s *^expression in mouse cerebellum and HEK293 cells induced robust increases in a broad spectrum of cell cycle-related genes. Both NAD-dependent and Pttg1-dependent pathways were responsible for mediating different subsets of these alterations, also incorporating changes in VCP/p97 localization and Ube1 expression. Cell proliferation rates were not modified by *Wld*^*s*^, suggesting that later mitotic phases of the cell cycle remained unaltered. We also demonstrate that *Wld*^*s *^concurrently altered endogenous cell stress pathways.

**Conclusion:**

We report a novel cellular phenotype in cells with altered neuronal vulnerability. We show that previous reports of diverse changes occurring downstream from *Wld*^*s *^expression converge upon modifications in cell cycle status. These data suggest a strong correlation between modified cell cycle pathways and altered vulnerability of axonal and synaptic compartments in postmitotic, terminally differentiated neurons.

## Abstract 2

Profiling of gene expression changes in mice harbouring the neurodegenerative Wlds mutation shows a strong correlation between changes in cell cycle pathways and altered vulnerability of terminally differentiated neurons.

## Background

Recent studies have highlighted the important role that vulnerability of nonsomatic neuronal compartments such as axons and synapses plays in the instigation and progression of neurodegenerative diseases, including Alzheimer's disease, multiple sclerosis, prion disease, Huntington's disease, and motor neuron diseases [1-4]. However, our understanding of the independent mechanisms that are required to regulate degenerative pathways in axons and synapses remains in its infancy. One powerful experimental tool that has already yielded novel insights into such pathways is the slow Wallerian degeneration (*Wld*^*s*^) mutation that selectively protects axons and synapses in the central and peripheral nervous systems following a wide variety of traumatic and disease-related, degeneration-inducing stimuli [5-12].

The *Wld*^*s *^mutation occurred spontaneously in a breeding colony of C57Bl/6 mice, resulting in a tandem triplication of an 85 kilobase region on distal chromosome 4 [13]. The *Wld*^*s *^gene encodes a fusion protein that comprises the full length of nicotinamide mononucleotide adenylyltransferase 1 (Nmnat1; a nicotinamide adenine dinucleotide [NAD^+^] synthesizing enzyme), coupled by a unique 18-amino-acid sequence to the amino-terminal 70 amino acids of the ubiquitination enzyme ubiquitination factor E4B (Ube4b) [14]. Transgenic expression of the *Wld*^*s *^gene is sufficient to confer the full neuroprotective phenotype in several species, including mice, rats, and *Drosophila *[14-16]. Despite providing substantial protection for axons and synapses, cell bodies are not protected in *Wld*^*s *^mice [17-19].

The Wld^s ^protein product appears to be localized exclusively to neuronal nuclei, suggesting that it confers its neuroprotective effects indirectly via modification of endogenous cellular pathways [14,20-22], but there remains considerable controversy over which cellular pathways may need to be targeted to confer *Wld*^*s*^-mediated neuroprotection. For example, several studies have demonstrated that the NAD/Sirt1 pathway can modulate axonal degeneration as a result of increased NAD levels, driven by Nmnat1 in the chimeric *Wld*^*s *^gene [23-25]. However, NAD pathways alone are insufficient to confer the full neuroprotective phenotype *in vivo *[26,27]. Other studies have suggested that modifications of the ubiquitin-proteasome system are required for neuroprotection, in part because of the ability of *Wld*^*s *^to bind valosin-containing protein (VCP/p97) [28,29]. Genomic and proteomic studies have identified other downstream effects of *Wld*^*s *^expression *in vivo *and *in vitro*. For example, array experiments have revealed modified expression levels for a range of genes, including the robust downregulation of mRNA encoding pituitary tumor transforming gene 1 (Pttg1 [22,30]). Similarly, proteomic experiments have demonstrated modifications in the levels of mitochondrial and/or synaptic proteins such as ubiquitin-activating enzyme E1 (Ube1) [31]. However, a unified hypothesis that brings together these distinct observations is currently lacking.

We made the previously unrecognized observation that many of these downstream changes also influence cell cycle. For example, Pttg1 is an oncogene with a recently established role in regulating the G_1 _to S phase transition of cell cycle [32]. Similarly, Ube1 is a protein with well established roles in cell cycle [33-36], and VCP/p97 localization is intricately linked to the cell cycle, with nuclear localization only occurring during late G_1 _phase [37]. In addition, several studies have demonstrated that NAD-dependent pathways play important roles in regulating cell cycle [38-40]. Taken together with numerous published studies reporting that cell cycle status can play an important role in modulating neuronal vulnerability and neurodegenerative pathways [41-49], these observations suggest that cell cycle modulation may provide a unified, common pathway on which genetic and proteomic changes downstream of *Wld*^*s *^may act to confer neuroprotection.

Here we show that *Wld*^*s *^expression in both mouse cerebellum *in vivo *and in HEK293 cells *in vitro *leads to robust increases in expression of a broad spectrum of cell cycle related genes, indicative of an attempt to re-enter cell cycle. We also provide evidence that these cell cycle changes involve all of the *Wld*^*s*^-mediated pathways detailed above (Pttg1, Ube1, NAD, and VCP), pushing postmitotic, terminally differentiated neurons toward cell cycle re-entry without affecting later mitotic phases. These data have identified a novel cellular phenotype in *Wld*^*s*^-expressing cells, unifying several diverse observations to reveal modifications in cell cycle status with concurrent alterations in cell stress. We propose that there exists a strong correlation between modified cell cycle pathways and altered vulnerability of axonal and synaptic compartments in postmitotic, terminally differentiated neurons.

## Results

### Increased expression of cell cycle genes and proteins in Wld^s^-expressing cells *in vivo *and *in vitro*

**Figure 1:**
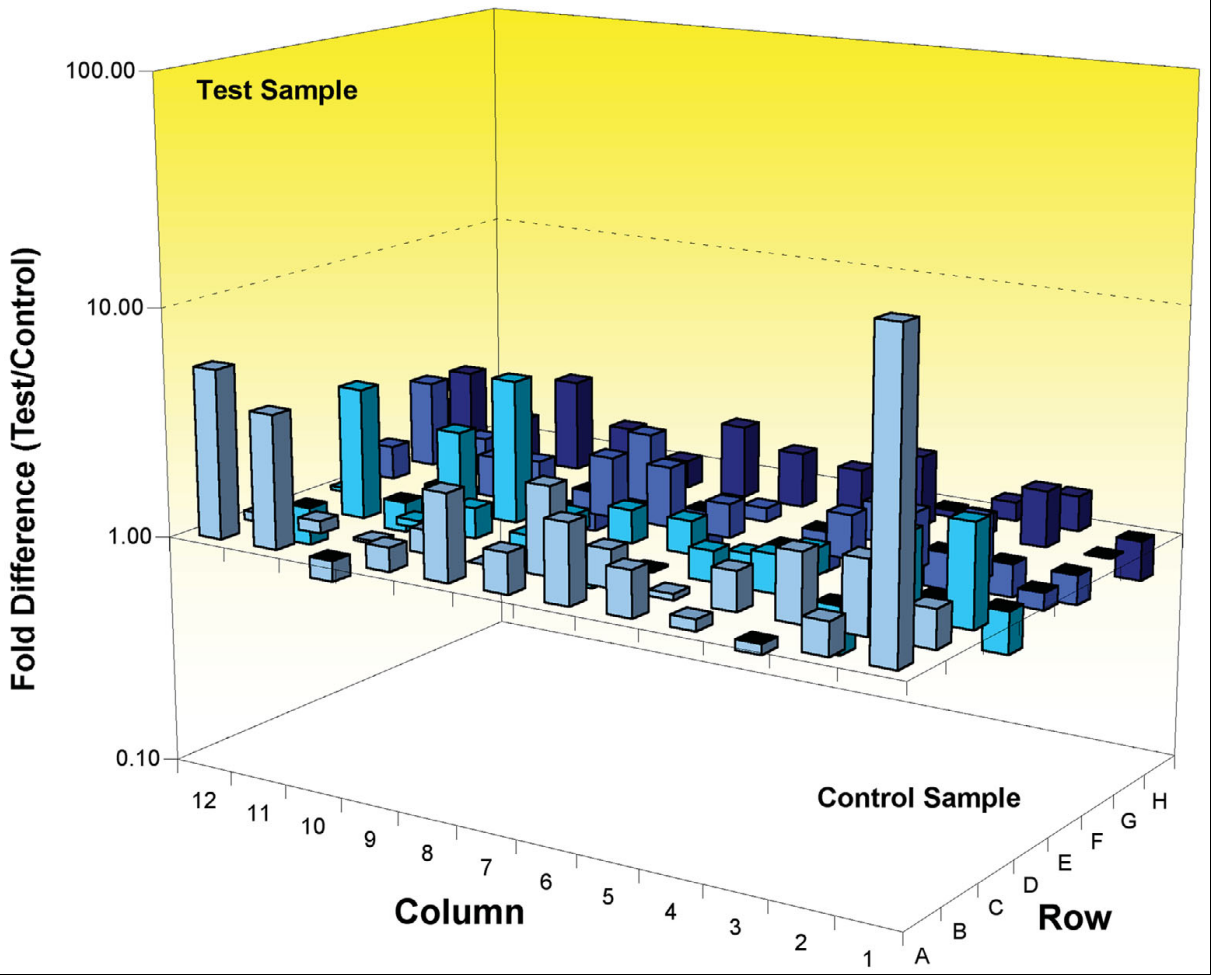
Up-regulation of cell cycle genes in terminally differentiated neurons from *Wld*^*s *^mouse cerebellum *in vivo*. Three-dimensional bar chart taken from SuperArray analysis software (cell cycle specific SuperArray; see Materials and methods) showing fold difference in expression levels for 84 cell cycle related genes, comparing wild-type cerebellum (control sample) with *Wld*^*s *^cerebellum (test sample). Individual genes with greater than twofold expression change can be found in Table 1.

We used cell cycle pathway-specific RT^2 ^profiler PCR arrays (see Materials and methods [below]) to quantify and compare the expression of cell cycle-related genes with high sensitivity. Initially, we used RNA extracted from the cerebellum of wild-type and *Wld*^*s *^mice because this tissue has proven ideal for comparative genomic experiments [22]. *Wld*^*s *^cerebellar granule cells are also known to express Wld^s ^protein at high levels and exhibit a strong neuroprotective phenotype [22]. We compared expression levels of 84 genes that regulate the cell cycle, including transitions between each of the phases, DNA replication, checkpoints, and arrest. Seventeen out of the 84 genes examined (around 20%) had expression levels increased by more than twofold in *Wld*^*s *^cerebellum (Figure 1 and Table 1). The array identified changes in genes associated with many different stages of the cell cycle rather than one specific stage (Table 1). Interestingly, no cell cycle related genes appeared to be suppressed greater than twofold by *Wld*^*s *^(Figure 1 and Table 1).

**Table 1:** Mouse SuperArray data showing greater than twofold cell cycle RNA expression changes in the cerebellum of *Wld*^*s *^mice compared with wild-type controls

Gene name	Symbol	Acc. Number	Array cell	Fold change	SD	Cell cycle function
V-abl Abelson murine leukemia oncogene 1	*Abl1*	NM_009594	A01	21.91	1.74	Regulation
Cyclin B1	*Ccnb1*	NM_172301	A12	5.50	0.65	M phase and regulation
Antigen identified by monoclonal antibody Ki 67	*Mki67*	XM_133912	D09	4.23	0.41	S phase and DNA replication
Cyclin A2	*Ccna2*	NM_009828	A11	3.85	0.65	Regulation
G protein-coupled receptor 132	*Gpr132*	NM_019925	C11	3.73	0.82	G_1 _phase and G_1_/S transition
Checkpoint kinase 1 homolog	*Chek1*	NM_007691	C01	2.74	0.81	G_2 _phase and G_2_/M transition
Transformation related protein 63	*Trp63*	NM_011641	G10	2.53	0.06	Negative regulator
Cyclin-dependent kinase 2	*Cdk2*	NM_016756	B07	2.43	0.52	M phase
Calcium/calmodulin-dependent protein kinase II, beta	*Camk2b*	NM_007595	A08	2.41	0.10	G_1 _phase and G_1_/S transition
S-phase kinase-associated protein 2 (p45)	*Skp2*	NM_013787	F12	2.40	0.26	G_1 _phase and G_1_/S transition and regulation
Wee 1 homolog	*Wee1*	NM_009516	G12	2.33	0.02	M phase
Meiotic recombination 11 homolog A	*Mre11a*	NM_018736	D10	2.28	0.57	S phase and DNA replication
CDC28 protein kinase 1b	*Cks1b*	NM_016904	C02	2.23	0.49	Checkpoint and arrest and regulation
Breast cancer 2	*Brca2*	NM_009765	A06	2.23	0.63	M phase and regulation and checkpoint and arrest
Cyclin C	*Ccnc*	NM_016746	B02	2.07	0.12	regulation
Transcription factor Dp 1	*Tfdp1*	NM_009361	G07	2.07	0.06	Regulation
SMT3 suppressor of mif two 3 homolog 1	*Sumo1*	NM_009460	G04	2.04	0.13	S phase and DNA replication
Retinoblastoma-like 2	*Rbl2*	NM_011250	F08	2.01	0.25	Negative regulator

SD, standard deviation.

**Figure 2:**
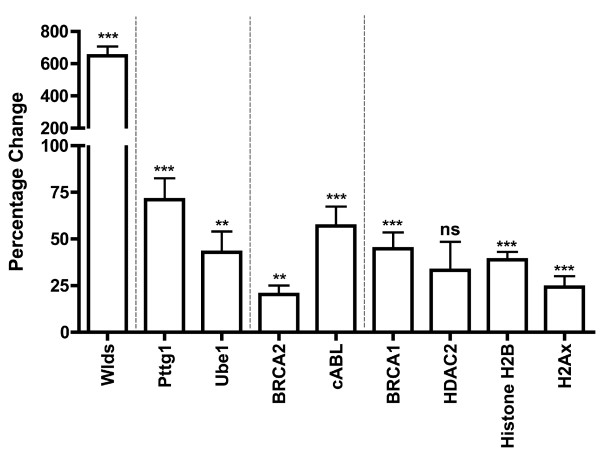
Quantitative fluorescent Western blots validate changes in cell cycle proteins in *Wld*^*s *^cerebellum *in vivo*. Bar chart showing percentage change in protein expression (mean ± standard error of the mean; n ≥ 3 for all proteins) in *Wld*^*s *^cerebellum compared with wild-type. As expected, Wld^s ^protein expression was highly upregulated (left bar). The second portion of the graph shows increases in both pituitary tumor transforming gene 1 (Pttg1) and ubiquitin-activating enzyme E1 (Ube1) proteins in *Wld*^*s *^mice, both of which have previously been implicated in the *Wld*^*s *^neuroprotective phenotype [22,31]. The third portion of the graph shows validation for two genes highlighted on the SuperArray analysis as being upregulated by more than twofold. The final portion of the graph shows similar increases in cell cycle proteins not included on the SuperArray plate, showing that increased expression of cell cycle proteins is not restricted to those included on the SuperArray. Statistical tests were carried out comparing raw expression data from wild-type mice with those from *Wld*^*s *^mice. **P < 0.01, *P *< 0.001 by unpaired *t*-test (two-tailed). ns, not significant.

To confirm that RNA changes led to corresponding changes in protein levels, we quantified protein expression levels in the cerebellum of *Wld*^*s *^and wild-type mice *in vivo*. We chose to focus on one of the genes with a large RNA change and one with a smaller change, just above the twofold threshold, where good antibodies were available (cABL and Brca2, respectively; Table 1). The protein product for both of these genes exhibited corresponding increased expression levels, of a similar ratio to that seen for RNA (Figure 2). In addition, we examined protein levels of other known cell cycle regulators to show that the changes observed on the PCR arrays were not exclusive. Three of the four additional proteins examined (histone H2B, BRCA1, and phosphohistone H2Ax) exhibited significantly increased expression levels in *Wld*^*s *^cerebellum, which is in keeping with the general trend observed on the PCR arrays (Figure 2).

**Figure 3:**
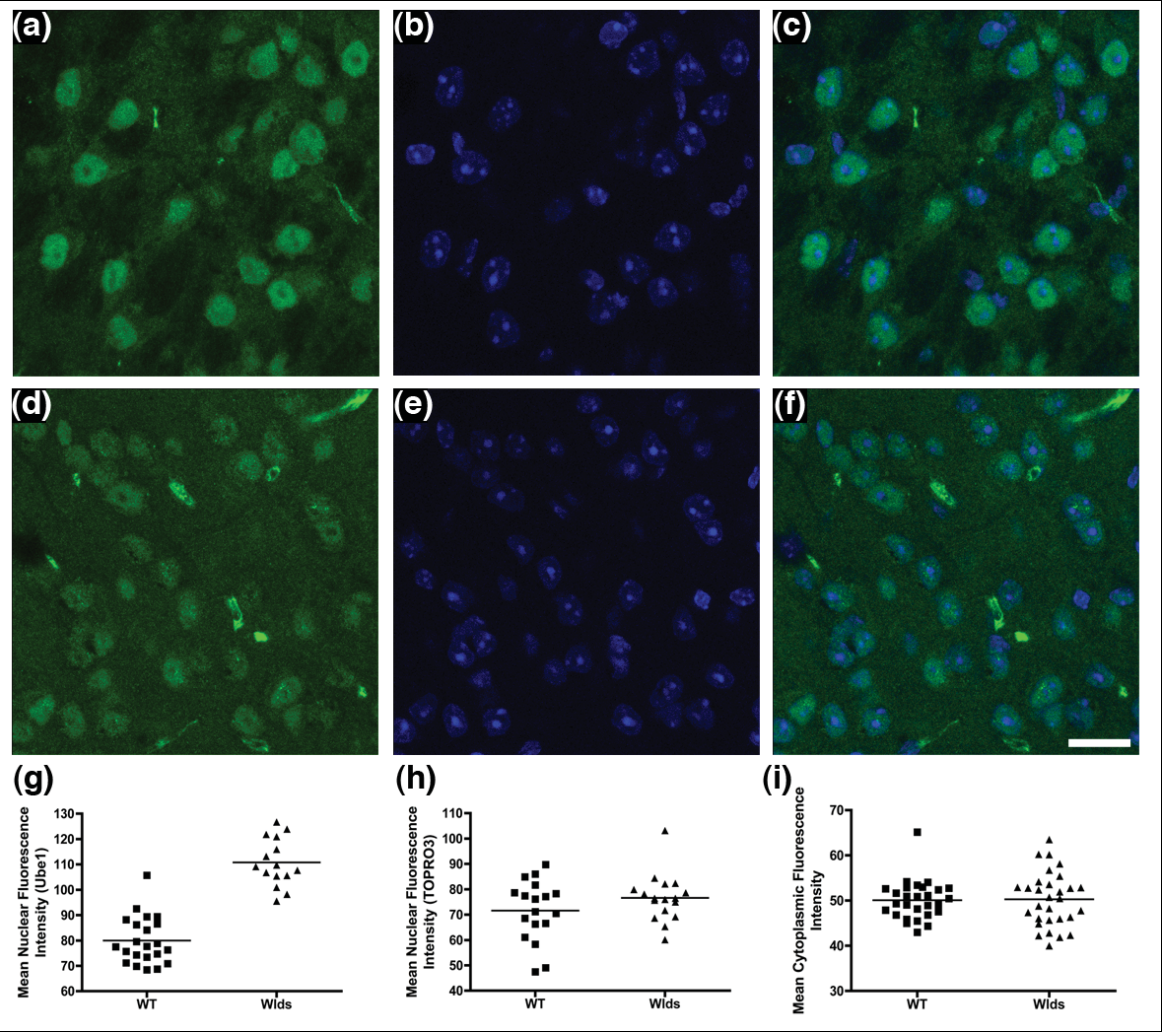
Immunocytochemistry confirms increased nuclear expression of Ube1 in *Wld*^*s *^mouse cerebellum. Confocal micrographs of cerebellar granule cells from **(a-c) ***Wld*^*s *^and **(d-f) **wild-type mice. Ubiquitin-activating enzyme E1 (Ube1) is shown in green and the nuclear marker TOPRO3 is shown in blue (panels a and d show Ube1; panels b and e show TOPRO3; and panels c and f show both markers). Note how Ube1 protein appears to be more strongly expressed in the nuclei of *Wld*^*s *^cerebellar neurons, whereas TOPRO3 and cytoplasmic levels of Ube1 appear unchanged. **(g-i) **Scatter plots (line indicates mean) of fluorescence intensity (see Materials and methods) of nuclear Ube1 (panel g), nuclear TOPRO3 (panel h), and cytoplasmic Ube1 (panel i). Only nuclear Ube1 was significantly increased in intensity in *Wld*^*s *^neurons (*P *< 0.001; by unpaired, two-tailed *t*-test). Scale bar 20 μm.

Next, we established that protein levels of two other cell cycle regulators, not included on the PCR array chip but previously shown to be modified in *Wld*^*s *^neurons, were similarly altered. Previous studies have demonstrated that protein levels of Ube1 (a protein with known cell cycle involvement [33-36]) are increased in *Wld*^*s *^synapses [31], and we were able to confirm this finding by showing increased total Ube1 protein levels in *Wld*^*s *^cerebellum (Figure 2). In addition, immunocytochemical staining for Ube1 confirmed increased nuclear expression levels in *Wld*^*s*^-expressing neurons *in vivo *(Figure 3). We also found that Pttg1 protein levels (another protein that regulates cell cycle pathways [32]) were significantly increased in *Wld*^*s *^cerebellum (Figure 2), which is in keeping with changes in all other cell cycle regulators modified by *Wld*^*s*^. This result was surprising because although Pttg1 protein levels had not previously been examined in *Wld*^*s*^-expressing cells, several previous reports have identified reduced mRNA levels for Pttg1 [22,30].

To verify that the alterations in cell cycle gene expression were occurring as a direct result of the presence of *Wld*^*s*^, and to further confirm that RNA changes observed in *Wld*^*s *^mouse cerebellum led to corresponding changes in protein levels, we examined the effects of *Wld*^*s *^on cell cycle in human embryonic kidney (HEK293) cells after transfection with enhanced green fluorescent protein (eGFP)-tagged *Wld*^*s *^constructs [22]. We selected HEK293 cells for our experiments for two main reasons. First, we wanted to consider whether the expression changes observed in mouse neurons *in vivo *could be replicated in a human cell line, as has previously been demonstrated for other *Wld*^*s*^-mediated changes in gene expression [22]. Second, HEK293 cells are an experimentally amenable, homogenous cell line that is routinely used to study transcriptional effects [22,50] and to model degenerative mechanisms in the human nervous system [51,52].

**Figure 4:**
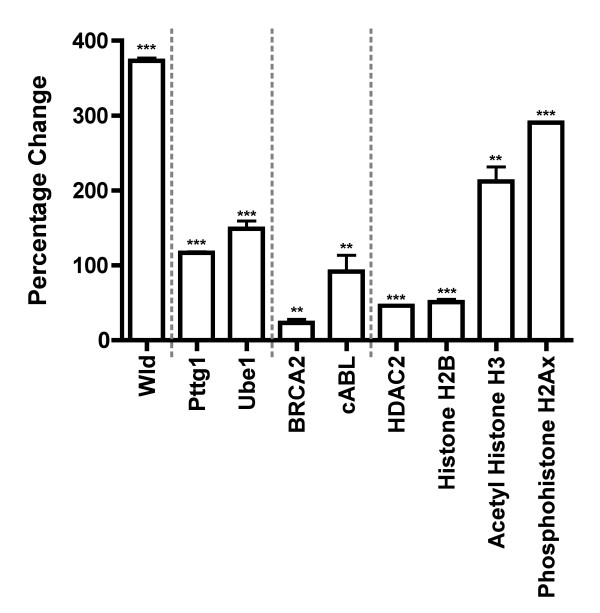
Quantitative fluorescent Western blots validate changes in cell cycle proteins in *Wld*^*s*^-expressing HEK293 cells *in vitro*. Bar chart showing percentage change in protein expression (mean ± standard error of the mean; n ≥ 3 for all proteins) in *Wld*^*s*^-transfected HEK293 cells compared with enhanced green fluorescent protein (eGFP)-transfected control cells. As expected, Wld^s ^protein expression was highly upregulated (left bar). The second portion of the graph shows increases in both pituitary tumor transforming gene 1 (Pttg1) and ubiquitin-activating enzyme E1 (Ube1) proteins following *Wld*^*s *^transfection, both of which were previously implicated in the *Wld*^*s *^neuroprotective phenotype [22,31]. The third portion of the graph shows validation for two genes highlighted on the SuperArray analysis as being upregulated by more than twofold. The final portion of the graph shows similar increases in cell cycle proteins not included on the SuperArray plate, showing that increased expression of cell cycle proteins is not restricted to those included on the SuperArray. All genes were significantly increased in expression levels in *Wld*^*s*^-transfected cells compared with control cells. **P < 0.01, ***P < 0.001 by unpaired *t*-test (two-tailed).

As for the cerebellar experiments, we again chose initially to focus on one gene with a large RNA change (Abl1) and one with a change just above the twofold threshold (Brca2). The protein product for both of these genes exhibited corresponding increased expression levels, of a similar ratio to that seen for RNA (Figure 4). In addition, we once again examined protein levels of other known cell cycle regulators to show that the changes observed on the PCR arrays were not exclusive. All four additional proteins examined (HDAC2, histone H2B, acetyl histone H3, and phosphohistone H2Ax) showed increased expression levels in *Wld*^*s*^-transfected cells, which is in keeping with the general trend observed on the PCR arrays (Figure 1). These experiments also provided further confirmation that both Ube1 and Pttg1 protein levels are increased by *Wld*^*s *^expression (Figure 4; compare with Figures 2 and 3).

**Figure 5:**
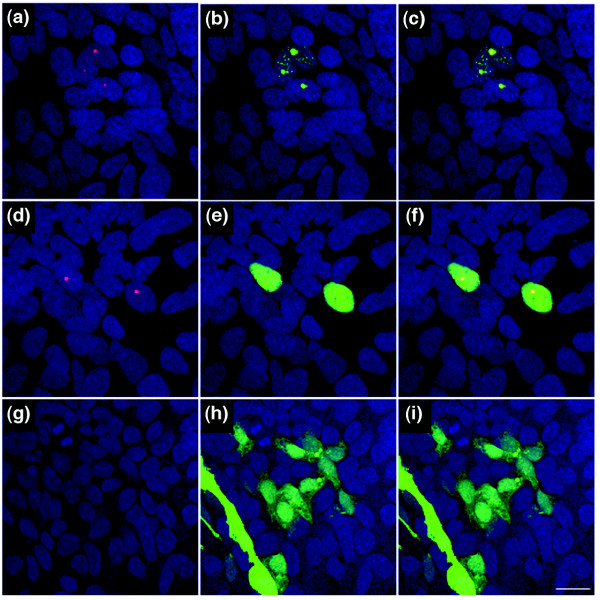
Increased expression of the cell cycle marker phosphohistone H2Ax in Wld^s ^transfected HEK293 cells. Confocal micrographs of HEK293 cells 5 days after transfection with either an **(a-f) **enhanced green fluorescent protein (eGFP)-*Wld*^*s *^construct or **(g-i) **a eGFP-only control construct. Immunocytochemical labeling of phosphohistone H2Ax is shown in red, the nuclear marker TOPRO3 is shown in blue, and constructs are expressing in green (panels a, d and g show H2Ax and TOPRO3; panels b, e and h show construct and TOPRO3; and panels c, f and i show all three markers). Note how phosphohistone H2Ax protein can only be seen in nuclear puncta where *Wld*^*s *^is being expressed. Note that not all cells have transfected with construct, and non-*Wld*^*s *^expressing cells identifiable by their TOPRO3 labeled nuclei do not have corresponding H2Ax puncta. H2Ax puncta were found in all *Wld*^*s*^-expressing cells, regardless of the nuclear distribution of *Wld*^*s *^(panels a to c show *Wld*^*s *^in nuclear inclusions; panels d to f show *Wld*^*s *^expressed in a strong diffuse manner throughout the nucleus). Scale bar 10 μm.

Because the Wld^s ^protein is known to have a predominantly nuclear distribution [20,21], and most cell cycle proteins modulate cell cycle via interactions in the nucleus, we next tested whether *Wld*^*s *^expression altered the nuclear expression of cell cycle proteins. We chose to investigate the nuclear distribution of phosphohistone H2Ax in *Wld*^*s*^-transfected HEK293 cells because this protein has a well-established role in the cell cycle [53,54] and was among the largest protein changes identified in HEK293 cells (Figure 5; see Figures 2 and 4 for phosphohistone H2Ax protein levels *in vivo *and *in vitro*). Not all cells express *Wld*^*s *^using our transfection protocol, as identified by the presence of an eGFP signal (Figure 5b,e). We were therefore able to compare directly experimental cells expressing *Wld*^*s *^or eGFP-only controls with neighbouring nontransfected cells. Anti-phosphohistone H2Ax antibodies revealed intense nuclear spots of phosphohistone H2Ax in all cells expressing *Wld*^*s *^(Figure 5a-f). However, neighbouring cells not expressing *Wld*^*s *^did not show any phosphohistone H2Ax nuclear puncta. No phosphohistone H2Ax staining was observed in control cells transfected with eGFP, indicating that the response was not simply the result of a large accumulation of foreign protein in the nucleus (Figure 5g-i).

**Figure 6:**
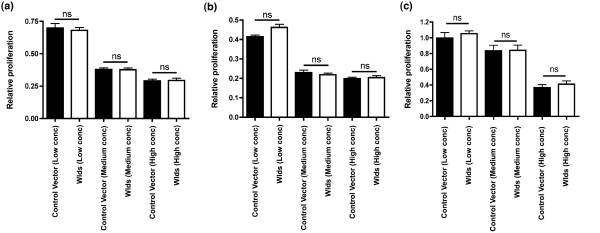
*Wld*^*s *^does not influence late stages of cell cycle regulating cell proliferation in NT2 cells. Bar charts showing relative proliferation rates of NT2 cells transfected with either a control vector (black bars) or a *Wld*^*s *^vector (white bars) at low, medium, and high concentrations. **(a) **Panel a shows no difference in proliferation at 48 hours after transfection using an MTT (3-[4,5-dimethylthiazolyl-2]-2,5-diphenyltetrazolium bromide) assay. **(b) **Panel b similarly shows no difference in proliferation at 72 hours after transfection using an MTT assay. **(c) **Panel c shows no difference in proliferation at 48 hours after transfection using a tritiated thymidine incorporation assay (all comparisons *P *> 0.05; analysis fo variance with Tukey's *post hoc *test).

Because we had found that a broad spectrum of cell cycle genes and proteins were modified by *Wld*^*s *^(Table 1), we next tested whether *Wld*^*s *^can influence neurons to pass through the complete cell cycle by quantifying proliferation rates in a human neuronal cell line (NT2 cells) using an MTT (3-[4,5-dimethylthiazolyl-2]-2,5-diphenyltetrazolium bromide) assay. Introduction of a *Wld*^*s *^construct into NT2 cells did not modify cell proliferation rates compared with vector-only transfected cells, either at 48 or 72 hours after transfection, or at low, medium, or high doses (Figure 6a-b). These findings were confirmed using tritiated thymidine uptake assays where values were normalized to low dose treatment (mean count: 14,770 ± 1,259 disintegrations per minute [DPM]; Figure 6c). Tritiated thymidine uptake assays were performed at 48 hours post-transfection in order to corroborate data from MTT assays generated at the same experimental time point and because this was the time point anticipated to give the maximum chance of detecting a proliferative change in these cells. These data suggest that *Wld*^*s *^upregulates the expression of a broad range of cell cycle regulators, pushing cells toward cell cycle re-entry, but that pathways influencing later stages of the cycle, such as mitotic cell division, remain inhibited.

**Figure 7:**
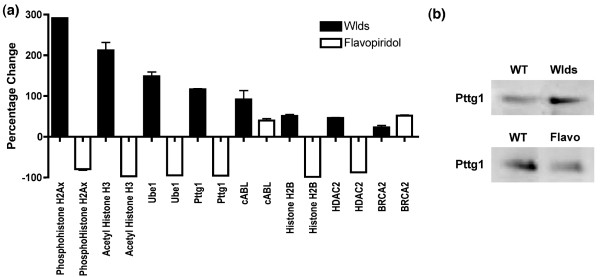
Pharmacological inhibition of cell cycle progression (flavopiridol) versus *Wld*^*s*^: opposing changes in cell cycle proteins. **(a) **Bar chart showing protein expression assayed by quantitative fluorescent western blots in HEK293 cells transfected with *Wld*^*s *^(black bars) or treated with exogenous flavopiridol (10 μmol/l; cell cycle inhibitor). Whereas *Wld*^*s *^induced increases in all cell cycle proteins, flavopiridol treatment led to decreased expression of the majority of proteins examined. **(b) **Representative Western blots showing pituitary tumor transforming gene 1 (Pttg1) protein levels in HEK293 cells comparing control versus *Wld*^*s *^transfected cells (top panel) and control versus flavopiridol treated cells (bottom panel). Note how Pttg1 protein levels are increased by *Wld*^*s *^expression and decreased by flavopiridol treatment.

In order to confirm that *Wld*^*s*^-mediated changes in cell cycle genes/proteins were pushing terminally differentiated neurons toward cell cycle re-entry rather than inhibiting cell cycle activation, we compared the profile of *Wld*^*s*^-mediated protein changes with changes induced by a well known pharmacologic inhibitor of the cell cycle: the cyclin-dependent kinase inhibitor flavopiridol. Treatment of HEK293 cells with flavopiridol at an established active concentration (10 μmol/l [48]) resulted in suppression of six out of eight cell cycle proteins that were increased in *Wld*^*s*^-transfected HEK293 cells (Figure 7). Thus, pharmacologic inhibition of the cell cycle also induced changes in cell cycle proteins known to be altered by *Wld*^*s*^, but importantly these changes in expression levels occurred in the opposite direction. These data confirmed that *Wld*^*s *^reactivates dormant cell cycle pathways, pushing cells toward cell cycle re-entry rather than inhibiting it.

### Role of Pttg1, NAD, and VCP pathways in mediating cell cycle modulation

**Figure 8:**
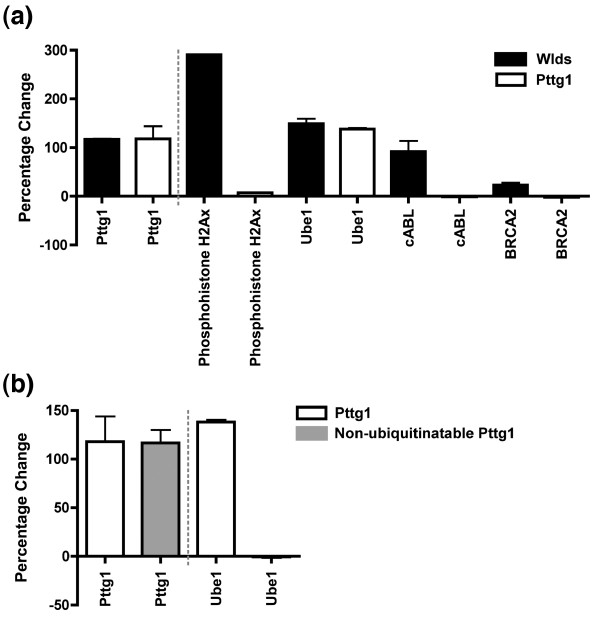
Over-expression of ubiquitinatable Pttg1 is required to elicit changes in the cell cycle protein Ube1. Presented are quantitative fluorescent Western blots of HEK293 cells (n = 3 for all proteins). **(a) **Changes in four cell cycle proteins known to be modified by *Wld*^*s *^after transfection with either a *Wld*^*s *^construct (black bars) or a pituitary tumor transforming gene 1 (Pttg1) over-expression construct (white bars). The first portion of the graph shows normalized Pttg1 levels accounting for differences in transfection efficiency. Note how Pttg1 induced the same level of increase in ubiquitin-activating enzyme E1 (Ube1) expression as *Wld*^*s *^but had no effect on the three other proteins. **(b) **changes in Ube1 can only be induced by a ubiquitinatable form of Pttg1, because transfection with a non-ubiquitinatable form of Pttg1 (gray bars) could not elicit any changes in Ube1 expression (right portion of graph).

After demonstrating that the *Wld*^*s *^gene robustly modifies cell cycle status in a variety of cell types *in vivo *and *in vitro*, we next investigated whether any of the previously identified downstream modifications induced by *Wld*^*s *^play a role in mediating cell cycle changes. First we investigated whether Pttg1 alone, as a known regulator of G_1 _to S phase cell cycle transition [32] with increased protein levels in *Wld*^*s*^-expressing cells (see Figures 2 and 4), was capable of mediating *Wld*^*s*^-induced effects on cell cycle proteins. We compared expression levels of four previously highlighted cell cycle proteins following transfection of HEK293 cells with either a *Wld*^*s *^construct [22] or a Pttg1 over-expression construct [55] (Figure 8a). Three of the four proteins examined were not modified by Pttg1 expression alone (Figure 8a), suggesting that other pathways are also required to induce the full range of cell cycle related changes (see below). However, Ube1 upregulation was induced by Pttg1 over-expression to a similar extent as seen with *Wld*^*s*^. This suggests that elevated Ube1 protein levels previously reported in *Wld*^*s *^synapses [31] are occurring downstream from increases in Pttg1 protein levels.

Pttg1 is currently the only known physiological substrate for the E4 ubiquitination factor Ube4b [56], which is one of the constituent parts of the chimeric *Wld*^*s *^gene [13]. In order to establish whether the ability of Pttg1 to be ubiquitinated is important for the regulation of Ube1, we repeated the experiment using an over-expression construct containing a non-ubiquitinatable form of Pttg1 [57]. The inability to be ubiquitinated completely abolished the ability of Pttg1 to increase Ube1 protein levels (Figure 8b), showing that ubiquitination of Pttg1 by Ube4b (and/or other proteins in the ubiquitin pathway) is likely to be important for *Wld*^*s*^-mediated cell cycle changes.

**Figure 9:**
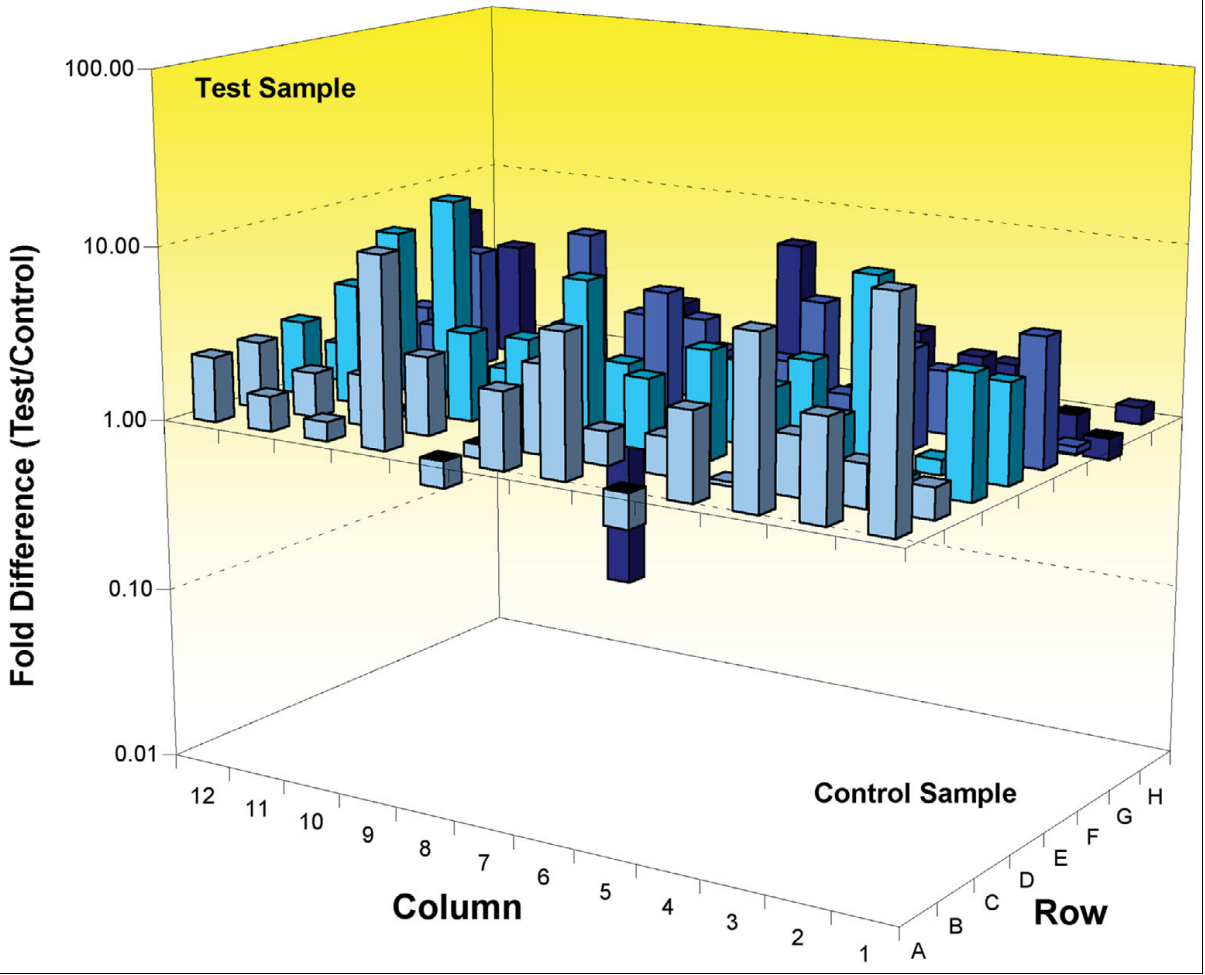
Upregulation of cell cycle genes in HEK293 cells treated with 1 mmol/l exogenous NAD. Three-dimensional bar chart taken from SuperArray analysis software (cell cycle SuperArray; see Materials and methods) showing fold difference in expression levels for 84 cell cycle related genes comparing vehicle treated HEK293 cells (control sample) with nicotinamide adenine dinucleotide (NAD) treated HEK293 cells (test sample). Individual genes with a greater than twofold expression change can be found in Table 2. NAD, nicotinamide adenine dinucleotide.

**Figure 10:**
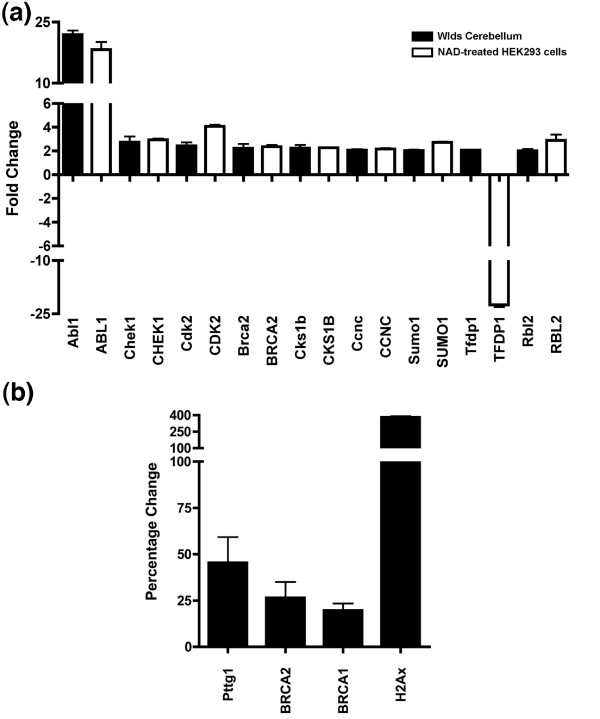
NAD-induced changes in cell cycle genes mimic *Wld*^*s*^-induced changes. **(a) **Bar chart showing greater than twofold changes in cell cycle genes from SuperArray experiments on *Wld*^*s *^cerebellum (black bars; see Table 1) compared with nicotinamide adenine dinucleotide (NAD) treated HEK293 cells (white bars; see Table 2). Of the nine genes examined, eight responded similarly in both experimental groups. **(b) **Bar chart showing percentage difference in protein expression in NSC34 cells treated with 1 mmol/l exogenous NAD as compared with control-treated cells.

Next, we investigated whether NAD-dependent pathways play a role in mediating cell cycle changes, because several recent studies have suggested that the Nmnat1 portion of the chimeric *Wld*^*s *^gene plays a significant role in conferring a neuroprotective phenotype by elevating NAD levels and increasing sirtuin activity [23-25]. To examine whether NAD pathways influence cell cycle changes observed in *Wld*^*s*^-expressing cells, we performed cell cycle pathway specific RT^2 ^profiler PCR arrays (using human rather than mouse arrays; see Materials and methods [below]) on HEK293 cells treated with 1 mmol/l NAD applied exogenously to the culture medium. This NAD treatment has previously been shown to confer axonal protection *in vitro *[23] and to mediate selected *Wld*^*s*^-induced transcriptional changes [22]. Forty-eight out of the 84 genes examined exhibited greater than twofold changes in expression after NAD treatment. In a similar result to that obtained in the *Wld*^*s *^mouse cerebellar array experiments, the vast majority (47 out of the 84) of modified genes had increased expression levels in the NAD treated cells (Figure 9 and Table 2). Only one cell cycle related gene appeared to be suppressed greater than twofold by NAD (Figure 9). A direct comparison of SuperArray data from *Wld*^*s *^cerebellum and NAD-treated HEK293 cells showed changes of a similar magnitude for eight out of the nine genes examined (Figure 10a; only nine candidate genes could be directly compared due to their presence/alteration on both arrays). Increases in protein expression levels of Pttg1, BRCA2, BRCA1, and H2Ax in NSC34 cells treated with 1 mmol/l NAD for 4 days confirmed that these NAD-induced changes extend beyond those included on the SuperArray, extend to the protein level, and can occur in neuronal cells (Figure 10b). These data suggest that elevated exogenous NAD levels can mimic many, but importantly not all, *Wld*^*s*^-induced cell cycle changes.

**Table 2:** Human SuperArray data showing greater than twofold cell cycle RNA expression changes in NAD-treated HEK293 cells compared with controls

Gene name	Symbol	Acc. Number	Array cell	Fold change	SD	Cell cycle function
V-abl Abelson murine leukemia viral oncogene homolog 1	*ABL1*	NM_005157	A01	18.25	3.19	S phase and DNA replication and regulation
Cullin 2	*CUL2*	NM_003591	D10	14.32	1.89	G_1 _phase and G_1_/S transition and checkpoint and arrest
B-cell CLL/lymphoma 2	*BCL2*	NM_000633	A09	12.44	2.12	Regulation
Cyclin-dependent kinase inhibitor 2B (p15, inhibits CDK4)	*CDKN2B*	NM_004936	D03	11.03	2.37	Checkpoint and arrest and negative regulator
Anaphase promoting complex subunit 4	*ANAPC4*	NM_013367	A03	9.17	0.27	G_2 _phase and G_2_/M transition and regulation
Cullin 3	*CUL3*	NM_003590	D11	8.46	0.65	G_1 _phase and G_1_/S transition and checkpoint and arrest
Cyclin-dependent kinase 5, regulatory subunit 1 (p35)	*CDK5R1*	NM_003885	C07	8.00	1.66	Regulation
RAD1 homolog	*RAD1*	NM_002853	F09	7.43	0.48	Checkpoint and arrest
SERTA domain containing 1	*SERTAD1*	NM_013376	G06	7.41	0.27	G_2 _phase and G_2_/M transition
Ataxia telangiectasia and Rad3 related	*ATR*	NM_001184	A06	6.51	0.36	Checkpoint and arrest
Ubiquitin-activating enzyme E1	*UBE1*	NM_003334	G12	6.48	1.19	S phase and DNA replication
Dynamin 2	*DNM2*	NM_004945	E01	5.38	0.37	G_2 _phase and G_2_/M transition
Cell division cycle 16 homolog	*CDC16*	NM_003903	C01	4.94	0.43	M phase
HUS1 checkpoint homolog	*HUS1*	NM_004507	E07	4.89	0.74	Checkpoint and arrest
Cyclin-dependent kinase 8	*CDK8*	NM_001260	C11	4.86	1.25	Regulation
RAD51 homolog (RecA homolog)	*RAD51*	NM_002875	F11	4.77	3.09	M phase
Menage a trois homolog 1, cyclin H assembly factor	*MNAT1*	NM_002431	F05	4.51	0.18	G_2 _phase and G_2_/M transition
Tumor protein p53 (Li-Fraumeni syndrome)	*TP53*	NM_000546	G11	4.40	1.48	Checkpoint and arrest
Cyclin-dependent kinase 2	*CDK2*	NM_001798	C05	4.07	0.24	Checkpoint and arrest and regulation
Anaphase promoting complex subunit 2	*ANAPC2*	NM_013366	A02	3.76	0.63	G_1 _phase and G_1_/S transition and regulation
Growth arrest and DNA-damage-inducible, alpha	*GADD45A*	NM_001924	E03	3.70	0.08	Checkpoint and arrest and regulation
Cyclin-dependent kinase inhibitor 1B (p27, Kip1)	*CDKN1B*	NM_004064	D01	3.65	0.25	G_1 _phase and G_1_/S transition and checkpoint and arrest
Cyclin-dependent kinase inhibitor 3	*CDKN3*	NM_005192	D04	3.36	0.09	G_1_+G_2 _phase and G_1_/S + G_2_/M transition and checkpoint and arrest
CDK5 regulatory subunit associated protein 1	*CDK5RAP1*	NM_016408	C08	3.32	0.06	G_2 _phase and G_2_/M transition
Cyclin F	*CCNF*	NM_001761	B07	3.22	0.20	M phase and regulation
Cyclin-dependent kinase 6	*CDK6*	NM_001259	C09	3.22	0.23	G_1 _phase and G_1_/S transition and regulation
DIRAS family, GTP-binding RAS-like 3	*DIRAS3*	NM_004675	A04	3.15	0.12	?
CHK1 checkpoint homolog	*CHEK1*	NM_001274	D05	2.95	0.15	Checkpoint and arrest
Nibrin	*NBN*	NM_002485	F07	2.89	0.03	Checkpoint and arrest
Retinoblastoma-like 2 (p130)	*RBL2*	NM_005611	G04	2.89	0.83	Negative regulator
Cell division cycle 34 homolog	*CDC34*	NM_004359	C04	2.86	0.14	G_1 _phase and G_1_/S transition and checkpoint and arrest
Cyclin G2	*CCNG2*	NM_004354	B09	2.81	0.18	Checkpoint and arrest
BCL2-associated X protein	*BAX*	NM_004324	A07	2.76	0.06	Negative regulator
Proliferating cell nuclear antigen	*PCNA*	NM_182649	F08	2.76	0.19	S phase and DNA replication
SMT3 suppressor of mif two 3 homolog 1	*SUMO1*	NM_003352	G08	2.73	0.10	S phase and DNA replication
Cyclin-dependent kinase inhibitor 1A (p21, Cip1)	*CDKN1A*	NM_000389	C12	2.67	0.08	Checkpoint and arrest and regulation
Hect domain and RLD 5	*HERC5*	NM_016323	E06	2.54	0.04	G_2 _phase and G_2_/M transition
Cyclin-dependent kinase 4	*CDK4*	NM_000075	C06	2.52	0.13	G_1 _phase and G_1_/S transition and regulation
G-2 and S-phase expressed 1	*GTSE1*	NM_016426	E05	2.49	0.10	G_2 _phase and G_2_/M transition
Cyclin T2	*CCNT2*	NM_001241	B12	2.41	0.16	G_2 _phase and G_2_/M transition and regulation
Breast cancer 2, early onset	*BRCA2*	NM_000059	A12	2.36	0.27	Checkpoint and arrest and regulation
Retinoblastoma-like 1 (p107)	*RBL1*	NM_002895	G03	2.31	0.04	Negative regulator
MCM5 minichromosome maintenance deficient 5	*MCM5*	NM_006739	F03	2.28	0.13	S phase and DNA replication
CDC28 protein kinase regulatory subunit 1B	*CKS1B*	NM_001826	D07	2.28	0.03	G_2 _phase and G_2_/M transition and regulation
Cell division cycle 20 homolog	*CDC20*	NM_001255	C03	2.24	0.17	Regulation
Cyclin C	*CCNC*	NM_005190	B03	2.16	0.14	Regulation
MAD2 mitotic arrest deficient-like 2	*MAD2L2*	NM_006341	E11	2.03	0.46	Checkpoint and arrest
Transcription factor Dp-1	*TFDP1*	NM_007111	G09	-22.50	1.04	Regulation

NAD, nicotinamide adenine dinucleotide; SD, standard deviation.

**Figure 11:**
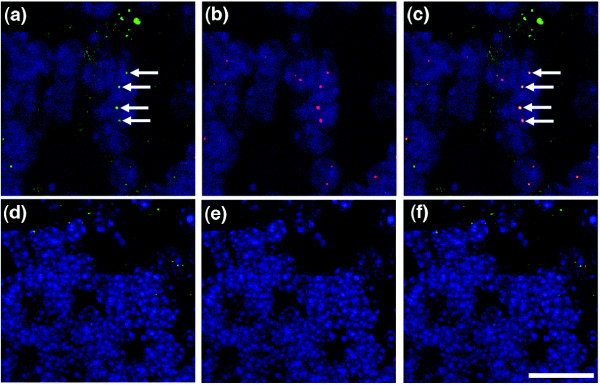
Increased nuclear expression of cell cycle marker VCP corresponding with Wld^s ^expression in mouse cerebellum. Confocal micrographs of cerebellar granule cells from **(a-c) ***Wld*^*s *^and **(d-f) **wild-type mice. Valosin-containing protein (VCP) is shown in green, the nuclear marker TOPRO3 is shown in blue, and Wld^s ^protein in red (panels a and d show VCP and TOPRO3; panels b and e show Wld^s ^and TOPRO3; and panels c and f show all three markers). Note how VCP protein can be seen in nuclear puncta with high frequency where *Wld*^*s *^is being expressed (arrows in panels a and c show four out of nine examples in this field of view). The majority of Wld^s ^puncta coincided with VCP puncta. Nuclear puncta of VCP were rarely observed in wild-type cerebellar granule cells. As expected, VCP was detectable as diffuse staining in the cytoplasm of neurons in both strains of mice. Scale bar = 20 μm.

Alongside identified changes in Pttg1/Ube1 expression and NAD pathways, previous studies have implicated VCP-mediated pathways (also known as p97 and CDC48) in *Wld*^*s*^-mediated neuroprotection, via its interaction with the Ube4b component of the Wld^s ^chimeric protein [28]. Moreover, VCP is known to be important in early stages of cell cycle progression; VCP is normally localised in the endoplasmic reticulum during nonproliferative states (for example, terminally differentiated neurons), but relocates to the nucleus during late G_1 _phase in a cell cycle dependent manner [37]. Thus, VCP localisation would not normally be observed in the nucleus of terminally differentiated neurons unless cell cycle had been reactivated and they are progressing toward S phase. To examine whether VCP redistribution associated with modified cell cycle status is modified by *Wld*^*s*^, we examined VCP localization in the cerebellum of *Wld*^*s *^and wild-type mice. These experiments revealed an expected cytoplasmic, non-nuclear localization in wild-type neurons, but distinct, strong nuclear puncta in most cerebellar granule cells in *Wld*^*s *^mice (Figure 11). As predicted from the finding that VCP binds *Wld*^*s *^[28], VCP localization in the nucleus was consistently found in the same nuclear puncta as Wld^s ^protein (Figure 11). These data provide further evidence that *Wld*^*s*^-expressing cells are being pushed toward the early phases of cell cycle re-entry and suggest that VCP binding may play a role in this process.

Thus, Pttg1/Ube1, NAD, and VCP pathways are all likely to be involved in mediating *Wld*^*s*^-induced modifications in cell cycle status. Taken together, these findings suggest that previous observations of apparently unrelated changes in gene and protein expression/activity downstream of *Wld*^*s *^can in fact be unified by their ability to modify the cell cycle.

### Modifications in cell stress pathways induced by *Wld*^*s *^*in vivo *and *in vitro*

**Figure 12:**
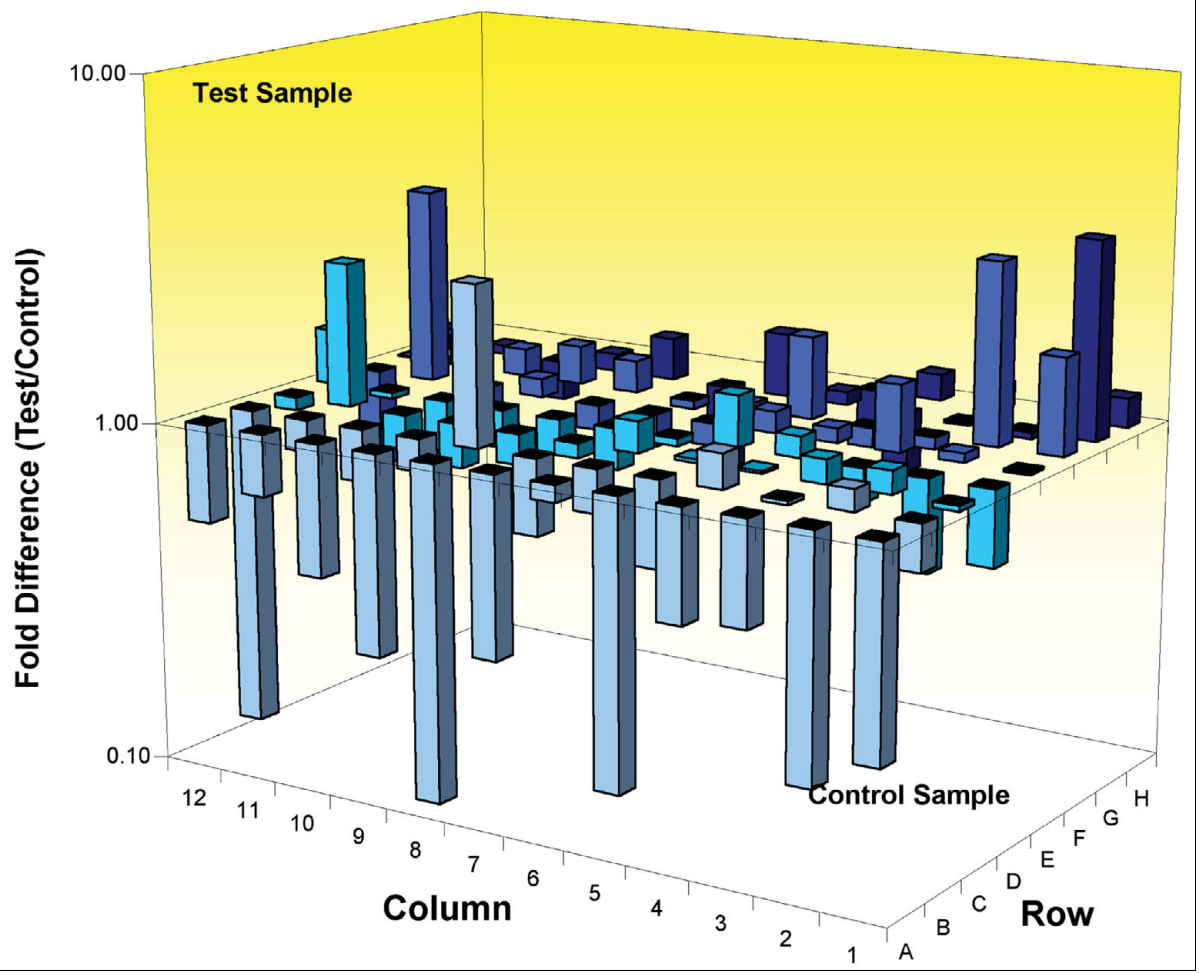
Widespread alterations in cell stress genes in uninjured/untreated *Wld*^*s *^mouse cerebellum *in vivo*. Three-dimensional bar chart taken from SuperArray analysis software (cell stress SuperArray; see Materials and methods) showing fold difference in expression levels for 84 cell stress related genes comparing wild-type cerebellum (control sample) with *Wld*^*s *^cerebellum (test sample). Individual genes with a greater than twofold expression change can be found in Table 3.

Changes in cell cycle status in terminally differentiated neurons are often associated with corresponding changes in cell stress pathways [58-60]. To examine whether cell stress pathways were also altered in *Wld*^*s*^-expressing cells, we used cell stress pathway-specific RT^2 ^profiler PCR arrays (see Materials and methods [below]) to compare mRNA levels in the cerebellum of wild-type and *Wld*^*s *^mice (Figure 12). Fourteen out of the 84 genes contained on the array were modified greater than twofold by *Wld*^*s*^, showing that a subset of cell stress pathways are also modified in *Wld*^*s *^(Figure 12 and Table 3). In contrast to the results from cell cycle arrays, however, *Wld*^*s *^neurons revealed both increases and decreases across a range of different cell stress proteins.

**Table 3:** Mouse SuperArray data showing greater than twofold cell stress RNA expression changes in the cerebellum of *Wld*^*s *^mice compared with wild-type controls

Gene name	Symbol	Acc. number	Array cell	Fold change	SD	Cell stress and toxicity function
Serine (or cysteine) peptidase inhibitor, clade E, member 1	*Serpine1*	NM_008871	G01	3.73	0.29	Inflammation
Interleukin 1 beta	*Il1b*	NM_008361	E11	3.69	0.82	Inflammation
Transformed mouse 3T3 cell double minute 2	*Mdm2*	NM_010786	F02	3.34	0.15	Growth arrest and senescence
Cytochrome P450, family 2, subfamily a, polypeptide 5	*Cyp2a5*	NM_007812	B08	2.92	0.48	Oxidative or metabolic stress
Fas ligand (TNF superfamily, member 6)	*Fasl*	NM_010177	C11	2.62	0.71	Apoptosis signaling
Bcl2-like 1	*Bcl2l1*	NM_009743	A04	-2.11	0.25	Apoptosis signaling
Cyclin C	*Ccnc*	NM_016746	A10	-2.44	0.46	Proliferation and carcinogenesis
Chemokine (C-C motif) ligand 21b	*Ccl21b*	NM_011124	A07	-3.37	0.25	Inflammation
Chemokine (C-C motif) ligand 4	*Ccl4*	NM_013652	A09	-3.86	0.40	Inflammation
Annexin A5	*Anxa5*	NM_009673	A01	-4.00	1.50	Apoptosis signaling
Ataxia telangiectasia mutated homolog (human)	*Atm*	NM_007499	A02	-4.99	0.70	DNA damage and repair
Caspase 1	*Casp1*	NM_009807	A05	-6.81	7.58	Apoptosis signaling
Cytochrome P450, family 3, subfamily a, polypeptide 11	*Cyp3a11*	NM_007818	B12	-8.60	0.52	Oxidative or metabolic stress
Chemokine (C-C motif) ligand 3	*Ccl3*	NM_011337	A08	-9.50	2.39	Inflammation

eGFP, enhanced green fluorescent protein; SD, standard deviation.

**Figure 13:**
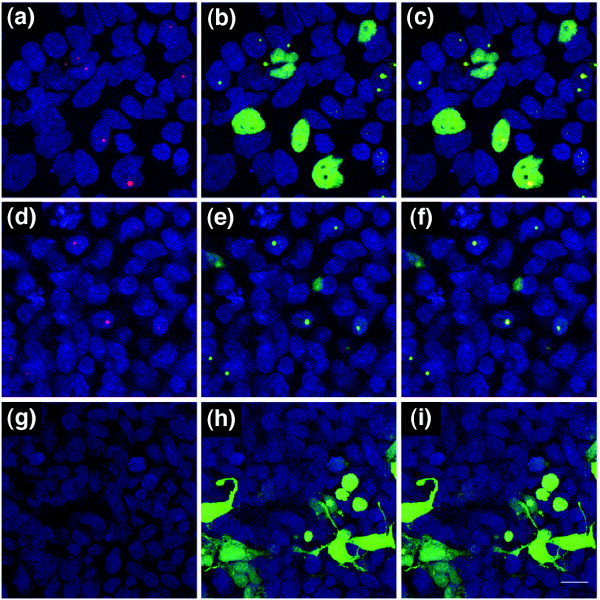
Increased nuclear expression of cell stress marker STI1 corresponding with Wld^s ^expression in HEK293 cells. Confocal micrographs of HEK293 cells transfected with either an **(a-f) **enhanced green fluorescent protein (eGFP)-*Wld*^*s *^construct or an **(g-i) **eGFP alone control construct. Stress induced phosphoprotein 1 (STI1) is shown in red, the nuclear marker TOPRO3 is shown in blue, and Wld^s ^protein in green (panels a, d and g show STI1 and TOPRO3; panels b, e and h show construct and TOPRO3; and c, f and i show all three markers). Note how STI1 protein can be seen in nuclear puncta with high frequency where *Wld*^*s *^is being expressed, but was never observed in non-*Wld*^*s*^-expressing cells. The majority of Wld^s ^puncta coincided with STI1 puncta. Nuclear puncta of STI1 were never observed in eGFP transfected control cells (panels g-i). Scale bar = 10 μm.

Finally, in order to confirm that *Wld*^*s *^altered nuclear localization, as well as expression, of cell stress proteins (as for cell cycle proteins shown in Figures 3 and 5), we investigated the expression and distribution of stress-induced phosphoprotein 1 (STI1) in *Wld*^*s*^-transfected HEK293 cells (Figure 13). We chose to use STI1 as a marker of cell stress *in vitro *in order to expand our coverage of cell stress modifications beyond those genes/proteins incorporated on the array chip and also because STI1 protein levels are known to be modified in *Wld*^*s *^synapses *in vivo *[31]. Anti-STI1 antibodies revealed nuclear spots of STI1 in most cells expressing *Wld*^*s *^(Figure 13a-f). However, neighbouring cells not expressing *Wld*^*s *^(because of less than 100% transfection efficiency) did not show any STI1 nuclear puncta. No STI1 staining was seen in control cells transfected with eGFP, indicating that stress responses were not simply occurring due to the presence of a large amount of foreign protein in the nucleus (Figure 13g-i). These findings were supported by data from quantitative Western blotting of STI1 protein levels in whole *Wld*^*s *^cerebellum *in vivo*, where STI1 levels were increased by 71.6 ± 6.8% (mean ± standard error of the mean; data not shown). Interestingly, we previously showed that STI1 protein levels are decreased in synapses protected by the *Wld*^*s *^gene *in vivo *[31]. The finding that nuclear STI1 immunoreactivity increases in *Wld*^*s *^transfected HEK293 cells suggests that some stress proteins may exhibit differential compartmental expression via redistribution within *Wld*^*s*^-expressing neurons, rather than simply having altered expression levels.

## Discussion

Here, we show that a strong correlation exists between modified cell cycle pathways and altered vulnerability of axonal and synaptic compartments in postmitotic, terminally differentiated neurons. We have demonstrated that the neuroprotective *Wld*^*s *^chimeric gene leads to a robust increase in expression of a broad spectrum of cell cycle-related genes in terminally differentiated neurons. These changes are indicative of an attempt to re-enter cell cycle in postmitotic neurons. Cell cycle alterations were identified in cerebellar neurons *in vivo *and could be replicated in HEK293 cell lines *in vitro*. We demonstrate that NAD, Pttg1/Ube1, and VCP pathways are all likely to be responsible for mediating distinct subsets of these downstream changes. Data from proliferation assays showing that *Wld*^*s *^does not alter cell division or proliferation rates suggests that terminally differentiated neurons expressing *Wld*^*s *^are pushed toward cell cycle re-entry, but do not go on to enter proliferation and growth phases. We also show that expression of the *Wld*^*s *^gene leads to modifications in endogenous cell stress pathways that are likely to result from modifications in cell cycle status.

Contrary to previous suggestions that *Wld*^*s*^-expressing neurons are 'normal', with the exception of a phenotype solely affecting axonal degeneration pathways [1], our experiments have revealed a novel cellular phenotype in *Wld*^*s*^-expressing cells: modifications in cell cycle status. This finding brings together diverse observations from several disparate studies investigating *Wld*^*s *^mechanisms (changes in Pttg1/Ube1, NAD, and VCP/p97 pathways), suggesting that modified cell cycle status might be a common endogenous pathway through which genomic and proteomic modifications downstream of *Wld*^*s *^can influence neuronal vulnerability.

### Pttg1/Ube1 pathways

Several studies have shown, using a range of experimental approaches and platforms, that *Wld*^*s *^robustly downregulates expression of Pttg1 mRNA [22,30]. Pttg1 plays a well established role in sister chromatid separation during mitosis, but recent data have identified an important additional role as a regulator of G_1 _to S phase cell cycle transition [32]. In the present study we showed that Pttg1 protein levels are significantly increased in *Wld*^*s*^-expressing cells. The most parsimonious explanation for the differences between protein and mRNA levels is that decreases in mRNA are generated by a compensatory, self-regulating feedback loop responding to elevated levels of Pttg1 protein. Because Pttg1 is the only known substrate for the Ube4b component of the *Wld*^*s *^gene [56], it is tempting to speculate that elevated Pttg1 protein levels result from abnormal ubiquitination and targeting for degradation, caused by *Wld*^*s*^-mediated alterations in the ubiquitin-proteasome pathway [28,29]. This finding also has implications for previous attempts to directly link Pttg1 to neuroprotection, because earlier studies examined neurodegenerative responses in Pttg1 null mice [22]. The current data suggest that repeating these experiments in Pttg1 over-expressing mice might reveal a neuroprotective phenotype, although the undoubted contribution made by other pathways (see below) suggests that Pttg1 over-expression alone would be unlikely to confer the full levels of *Wld*^*s*^-mediated neuroprotection.

We have also shown that increased Pttg1 protein levels induced by *Wld*^*s *^are responsible for mediating corresponding increases in expression of another cell cycle-related protein, namely Ube1. Thus, Pttg1 is likely to be a partial mediator of other cell cycle changes induced by *Wld*^*s*^. We previously identified increased protein levels of Ube1 in a population of striatal synapses from *Wld*^*s *^mice known to be protected from degeneration [31], suggesting that Ube1 may also play a role in directly modulating degenerative pathways in synaptic compartments of neurons. Importantly, we also found that the ability of Pttg1 to increase Ube1 protein levels was abolished if Pttg1 was expressed in a non-ubiquitinatable form. Because Pttg1 is the only known substrate for the Ube4b component of the *Wld*^*s *^gene [56], these data suggest that modified ubiquitination of Pttg1 by Ube4b (either in its native form or as part of the Wld^s ^protein) is likely to be required to mediate downstream changes in proteins such as Ube1. However, at this stage we cannot rule out the possibility that other proteins in the ubiquitin pathway alongside Ube4b are also responsible for mediating this response.

### NAD pathways

The most convincing evidence to date for the involvement of a single pathway in downstream mediation of the *Wld*^*s *^phenotype has come from studies showing that the Nmnat1 portion of the chimeric *Wld*^*s *^gene confers a neuroprotective phenotype via modulation of NAD levels [23-25]. Here, we have shown that experimental manipulation of NAD levels mimics many (but by no means all) of the cell cycle changes induced by *Wld*^*s*^. This finding raises the possibility that at least part of the NAD-mediated neuroprotective phenotype is generated by modulating cell cycle status. This suggestion is supported by several studies demonstrating that sirtuin-dependent pathways (which mediate the NAD neuroprotective phenotype [23]) play important roles in regulating cell cycle [38-40]. However, as with the Pttg1 findings discussed above, it is unlikely that NAD-mediated changes alone are sufficient to induce the full range of *Wld*^*s*^-mediated cell cycle changes, because NAD over-expression alone is not thought to be sufficient to confer the full neuroprotective phenotype *in vivo *or *in vitro *[26,27].

### VCP/p97 pathways

Alongside Pttg1 and NAD, pathways mediated by VCP (also known as p97 and CDC48) have also been shown to influence cell cycle [61]. For example, although VCP is predominantly a cytoplasmic protein, it is known to enter the nucleus during late G_1 _phase [37]. Here, we have shown that VCP, which is currently the only known binding partner for the Ube4b portion of *Wld*^*s *^[28,62], is localized to the nucleus in the majority of *Wld*^*s*^-expressing neurons. This is further corroborating evidence that cell cycle has been reactivated in terminally differentiated *Wld*^*s *^neurons and that they are progressing toward (or beyond) S phase. Although binding to VCP has not yet been demonstrated to be required for the *Wld*^*s *^phenotype, the present study suggests that if this is an important event, VCP may be acting via regulation of the cell cycle in a similar manner to Pttg1. Cell cycle events potentially attributable to VCP pathways detected in the current experiments include changes in expression levels of BRCA proteins that are known to interact with VCP in the nucleus [63].

### Cell cycle pathways and neurodegeneration

The hypothesis that *Wld*^*s *^may be modifying neurodegenerative pathways in axons and synapses via modulation of the cell cycle is in keeping with other literature on somatic neurodegeneration, in which cell cycle is known to influence vulnerability significantly. For example, it is now known that postmitotic, terminally differentiated neurons in the adult nervous system are not 'permanently postmitotic', but rather depend upon the constant suppression of cell cycle pathways to maintain their arrested status [41]. The ability to control cell cycle pathways is therefore a critical factor in stopping neurons entering a vulnerable state, where the risk for neurodegenerative mechanisms being instigated increases significantly [41-44]. Numerous examples of cell cycle regulation gone awry, modifying neuronal vulnerability, can be found in neurodegenerative conditions such as motor neuron disease, Alzheimer's disease, and stroke [45,46]. Furthermore, pharmacologic manipulation of cell cycle progression has been used to confer somatic neuroprotection in animal models of traumatic brain injury and stroke [47,48]. The current data suggest that the influence of cell cycle status on neuronal vulnerability is likely to extend beyond neurodegenerative mechanisms resident in cell soma to incorporate independent degenerative pathways in axonal and synaptic compartments. The *Wld*^*s *^gene may therefore provide an important experimental tool for future investigations into pathways through which cell cycle status modulates neuronal vulnerability.

The current data are also likely to be important for interpreting previous and future studies concerning *Wld*^*s*^-mediated neuroprotection both *in vivo *and *in vitro*. Because endogenous cell cycle and cell stress pathways are robustly modified by *Wld*^*s *^expression, it is difficult to imagine that *Wld*^*s*^-expressing cells do not have any other covert cellular phenotypes alongside neuroprotection. These may introduce additional variables that could conceivably alter experimental outcomes (for example, comparing Nmnat over-expressing cells *in vivo *with cells exposed to exogenous NAD *in vitro *[23-27]).

Current opinion suggests that cell cycle re-entry is damaging to neurons, whereas blocking cell cycle decreases vulnerability [41-44]. The finding that the neuroprotective *Wld*^*s *^gene pushes neurons toward cell cycle re-entry therefore appears at odds with this hypothesis. There are two possible explanations for this discrepancy. First, it is possible that the basic principle of re-entry is bad/suppression is good may not hold for all neurodegenerative pathways. Second, and perhaps more plausibly, it is possible that *Wld*^*s *^acts to 'prime' the cell against future neurodegenerative insults by inducing early-stage cell cycle changes - and cell stress modifications - without going as far as affecting proliferation and growth stages. This potential mechanism of action is in keeping with a known role for preconditioning, sublethal 'priming' events in conferring neuroprotection by modifying endogenous stress pathways [64-67].

### Cell stress pathways and *Wld*^*s*^

Our finding that cell stress pathways are also modified in *Wld*^*s *^mice, suggesting a possible 'primed state' of *Wld*^*s *^neurons, is in keeping with the findings of other recent studies. For example, it was recently demonstrated that the NMNAT1 component of the chimeric *Wld*^*s *^gene has functions alongside those involving NAD, acting as a chaperone for stress-response proteins such as heat shock protein-70 [68] (Wishart TM, Gillingwater TH, unpublished observations). In addition, we have recently shown that the mitochondrial proteome is modified at a basal level in protected *Wld*^*s *^synapses [31], suggesting intrinsic differences in the ability to respond to cell stress stimuli. This hypothesis has received experimental support from another recent study showing that NMNAT1 can protect against mitochondrial and oxidative stress [69].

Our study has identified possible individual stress-related proteins that may play an important role in the cell stress response in *Wld*^*s *^neurons. Interestingly, several of these proteins have already been implicated in other neuroprotective situations. For example, STI1 - shown in previous work [31] and the present study to have altered levels and subcellular localization in *Wld*^*s*^-expressing cells - appears to play an important role in neuroprotection and neuritogenesis [70] as well as in cell proliferation [71,72]. Further investigations into the *in vivo *role played by individual cell stress proteins modified by *Wld*^*s*^, such as STI1, in modulating neuronal phenotypes may therefore provide important insights into mechanisms underlying axonal and synaptic vulnerability.

## Conclusion

We have identified a strong and robust correlation between modified cell cycle pathways and altered vulnerability of axonal and synaptic compartments in terminally differentiated neurons by showing that the neuroprotective *Wld*^*s *^gene modifies cell cycle and cell stress status *in vivo *and *in vitro*. We conclude that *Wld*^*s*^-expressing cells have a potentially important, previously unreported cellular phenotype that is characterized by reactivation of normally suppressed cell cycle pathways in terminally differentiated neurons. We propose that multiple NAD-, Pttg1/Ube1-, and VCP-dependent pathways are likely to be required to modulate these cell cycle changes. The data suggest that further investigations into the role of cell cycle and cell stress status induced by *Wld*^*s *^are likely to provide insights into mechanisms that regulate axonal and synaptic degeneration in neurodegenerative disease.

## Materials and methods

### Mouse tissue and cell lines/treatments

Natural mutant C57Bl6/Wld^S ^(*Wld*^*S*^) mice and C57Bl/6 (wild type) mice (all aged 6 to 8 weeks) were obtained from Harlan Olac Laboratories (Bicester, UK) and housed within the animal care facilities in Edinburgh. Mice (minimum three mice per experimental group) were killed by cervical dislocation and the cerebellum was rapidly removed.

HEK293 and NSC34 (a mouse motor neuron-like cell line [73]) cells were maintained in Dulbecco's modified Eagle's medium with 10% fetal bovine serum and 1% penicillin/streptomycin (Invitrogen, Carlsbad, CA, USA) at 37°C in 5% carbon dioxide. For transfection with eGFP-*Wld*^*s *^[22], Pttg1 [55], and non-ubiquitinatable Pttg1 [57], 5 mg of the DNA was mixed with 10% (vol/vol) CaCl_2_. An equal volume of N,N-bis(2-hydroxyethyl)-2-aminoethanesulfonic acid was added and the solution gently dropped onto the HEK293 cell culture [22]. For some experiments, 1 mmol/l NAD (Sigma-Aldrich, Gillingham, UK) or 10 μmol/l Flavopiridol (obtained through the National Cancer Institute, Rockville, MD, USA) was added to the medium. All cells were incubated for 4 to 5 days and were checked on a phase contrast microscope before proceeding to either immunocytochemistry/microscopy or extraction of protein and/or RNA (at least three cultures for all transfections and treatments).

### RNA and protein extraction

RNA was extracted from cerebella of age-matched and sex-matched mice, or HEK293 cells, in tri-reagent (Sigma) in accordance with the manufacturer's instructions, as previously described [22]. Protein was extracted from cerebella of age-matched and sex-matched mice, or HEK293 cells, in RIPA buffer with 10% protease inhibitor cocktail (Sigma) [31].

### Super arrays

Mouse cell cycle (PAMM-020A), cell stress (PAM-003A), and human cell cycle (PAHS-020A) focused pathway arrays (Tebubio Superarrays, Peterborough, UK) in 96-well plate format, compatible with an ABI 7000 real-time PCR machine, were used to assay gene expression changes (three comparisons for each array type). Samples were added to the reaction plates and signal amplification by PCR was carried out using a Sybr-Green '1 step qRT-PCR kit' (Invitrogen). Analysis was carried out using the Analysis Suite spreadsheet provided by Tebubio Superarrays. The absence of DNA contamination and efficiency of amplification was confirmed using the analysis software provided. Gene functions listed in Tables 1 to 3 were obtained from the SuperArray product specification sheets. Raw data for all of these array experiments can be found online [74].

### Quantitative Western blots

Cerebellar/cultured cell protein was separated by SDS-PAGE on 4% to 20% pre-cast NuPage 4% to 12% Bis Tris gradient gels (Invitrogen) and then transferred to PVDF membrane overnight. The membranes were then blocked using Odyssey blocking buffer (Li-COR Biosciences, Lincoln, Nebraska, USA) and incubated with primary antibodies as per manufacturers instructions (BRCA2, cAbl, CCL3 and E1 ubiquitin activating enzyme [Abcam, Cambridge, MA, USA]; anti-HDAC2 clone 3F3, anti-histone H2B and anti acetyl histone H3 [Lake Placid Biologicals, Lake Placid, NY, USA]; Pds1 Ab1 clone DCS280/Anti-Pttg1 [LabVision Corporation, Freemont, CA, USA]; and antiphosphohistone H2Ax [Upstate, Billerica, MA, USA]). Anti-Wld^s ^antibodies were a kind gift from Dr Michael Coleman and were used as previously described [20,21]. Odyssey secondary antibodies were added in accordance with the manufacturer's instructions (Goat anti rabbit IRDye 680 and Goat anti mouse IRDye 800). Blots were imaged using an Odyssey Infrared Imaging System (Li-COR Biosciences). The scan resolution of the instrument ranges from 21 to 339 μm, and in this study blots were imaged at 169 μm. Quantification was performed on single channels with the analysis software provided. Bands were identified according to their relative molecular weight, as detailed in the manufacturer's antibody notes. Bands were delineated using Odyssey software and the arbitrary fluorescence intensity calculated by the software. For each membrane, scans were carried out at three different intensities in order to minimize possible user error in determining correct scan intensities or over-saturation of the membrane. The average of these three separate scans (giving an *n *of 1 per membrane) was used for further analysis.

### NT2 cell proliferation assays

MTT assays were carried out in 96-well plates. One hundred micrograms of MTT was added to 6,000 cells per well of NT2 cells transfected with control (vector only) or *Wld*^*s *^constructs (200 ng [low dose], 600 ng [medium dose], and 1,200 ng [high dose]), and incubated for 3 hours. Media were then removed and formazan crystals dissolved in 50 μl dimethyl sulfoxide. The absorbance of the dimethyl sulfoxide was read at 545 nm to estimate cell number. For 3H-thymidine incorporation assays, 3 × 10^5 ^NT2 cells per well of a 24-well plate were transfected with control (vector only) or *Wld*^*s *^constructs, as detailed above, and proliferation assayed at 48 hours. Mitogenesis was estimated from the measurement of nuclear 3H-thymidine incorporation. Cells were incubated with 0.2 Ci 3H-thymidine (specific activity 70-95 Ci/mmol; Amersham Biosciences, Little Chalfont, UK) for the last 5 hours of culture incubation. Cells were then washed twice in ice-cold phosphate-buffered saline (PBS), followed by 1 ml cold 5% trichloroaceteic acid, and left on ice for 20 minutes. Then, 0.1 mol/l sodium hydroxide was added to the cells before transfer to 4 ml of scintillant. Radioactive counts were determined by scintillation counting. Experiments comprised four replicates each and were performed on at least two separate occasions.

### Immunocytochemistry

Immunofluorescence staining was performed on either freshly cut (20 μm or 100 μm) cerebellar slices or HEK293 cells, fixed in 4% paraformaldehyde (Fisher Scientific, Loughborough, UK). Slices or cultured cells were incubated overnight in serum blocker consisting of 4% bovine serum albumin (Sigma) and 0.5% Triton X-100 (Sigma) in PBS. In cerebellar slices, anti-Wld^s ^antibodies (1:500 dilution in serum blocking solution [8]) were applied overnight and, after washing with PBS saline, a TRITC-conjugated anti-rabbit secondary antibody (DAKO, Glostrup, Denmark) was applied overnight. Primary antibodies, including Ube1 (Abcam), VCP (Abcam), Sti1 (BD Transduction Laboratories, San Jose, CA, USA), and phosphohistone H2Ax (Upstate), were also used at concentrations specified by the manufacturers. Anti-rabbit/mouse FITC-conjugated secondary antibodies (DAKO) were used on cerebellar slices and anti-rabbit/mouse TRITC-conjugated secondary antibodies (DAKO) were used on HEK293 cells. Secondary antibody only controls were also carried out and confirmed the specificity of antibodies used (data not shown). Finally, cerebellar slices and cultured cells were then washed in PBS and incubated in TOPRO 3 (Molecular Probes, Carlsbad, CA, USA) for 10 minutes before mounting in Mowoil.

Staining was visualised on a laser scanning confocal microscope (BioRad Radiance 2000; BioRad, Hemel Hempsted, UK) and Z-series were merged using Lasersharp (BioRad) software.

### Data analysis

All non-SuperArray data were collected in Microsoft Excel and all statistical analyses and graphs were produced using GraphPad Prism. Quantification of cytoplasmic and nuclear Ube1/TOPRO3 fluorescence was undertaken on confocal micrographs captured using identical microscope settings between images and specimens. No image manipulation was undertaken before quantification using standard fluorescence intensity tools in Image J software. Images were prepared for presentation in Adobe Photoshop.

## Abbreviations

eGFP, enhanced green fluorescent protein; MTT, 3-(4,5-dimethylthiazolyl-2)-2,5-diphenyltetrazolium bromide; NAD, nicotinamide adenine dinucleotide; Nmnat1, nicotinamide mononucleotide adenylyltransferase 1; PBS, phosphate-buffered saline; PCR, polymerase chain reaction; Pttg1, pituitary tumor transforming gene 1; STI1, stress-induced phosphoprotein 1; Ube1, ubiquitin-activating enzyme E1; Ube4b, ubiquitination factor E4B; VCP, valosin-containing protein; *Wld*^*s*^, slow Wallerian degeneration.

## Authors' contributions

TMW and THG conceived, designed and coordinated the study, undertook the genomic, proteomic and immunocytochemical assays and drafted the manuscript. HNP, SRJ, and CJM carried out the cell proliferation assays. All authors read and approved the final manuscript.
